# Validity of Capsule Endoscopy in Monitoring Therapeutic Interventions in Patients with Crohn’s Disease

**DOI:** 10.3390/jcm7100311

**Published:** 2018-09-29

**Authors:** Masanao Nakamura, Takeshi Yamamura, Keiko Maeda, Tsunaki Sawada, Yasuyuki Mizutani, Takuya Ishikawa, Kazuhiro Furukawa, Eizaburo Ohno, Hiroki Kawashima, Ryoji Miyahara, Anastasios Koulaouzidis, Yoshiki Hirooka, the Nagoya University Crohn’s Disease Study Group

**Affiliations:** 1 Department of Gastroenterology & Hepatology, Nagoya University Graduate School of Medicine, 65 Tsuruma-cho, Showa-ku, Nagoya 466-8550, Japan; y-mizu@med.nagoya-u.ac.jp (Y.M.); ishitaku@med.nagoya-u.ac.jp (T.I.); kazufuru@med.nagoya-u.ac.jp (K.F.); eono@med.nagoya-u.ac.jp (E.O.); h-kawa@med.nagoya-u.ac.jp (H.K.); myhr@med.nagoya-u.ac.jp (R.M.); 2 Department of Endoscopy, Nagoya University Hospital, Nagoya 466-8550, Japan; tyamamu@med.nagoya-u.ac.jp (T.Y.); kmaeda@med.nagoya-u.ac.jp (K.M.); t.sawada@med.nagoya-u.ac.jp (T.S.); hirooka@med.nagoya-u.ac.jp (Y.H.); 3 Endoscopy Unit, The Royal Infirmary of Edinburgh, Edinburgh EH16 4SA, UK; akoulaouzidis@hotmail.com

**Keywords:** capsule endoscopy, Crohn’s disease, mucosal healing, small bowel

## Abstract

Mucosal healing in Crohn’s disease (CD) can be evaluated by capsule endoscopy (CE). However, only a few studies have utilized CE to demonstrate the therapeutic effect of medical treatment. We sought to evaluate the validity of using CE to monitor the effect of medical treatment in patients with CD. One hundred (*n* = 100) patients with CD were enrolled. All patients had a gastrointestinal (GI) tract patency check prior to CE. Patients with baseline CE Lewis score (LS) ≤ 135 were included in the non-active CD group and ended the study. In those with LS > 135 (active CD group), additional treatment was administered, regardless of symptoms, as per the treating clinician’s advice. Patients of the active CD group underwent follow-up CE assessment 6 months later. Out of 92 patients with confirmed GI patency who underwent CE, 40 (43.4%) had CE findings of active inflammation. Of 29 patients with LS > 135 who received additional medications and underwent follow-up CE, improvement of the LS was noted in 23 (79.3%) patients. Eleven patients were asymptomatic but received additional medications; 8 (72.7%) had improvement of the LS. This study demonstrated that additional treatment even for patients with CD in clinical remission and active small-bowel inflammation on CE can reduce mucosal damage.

## 1. Introduction

The main goal in the treatment for Crohn’s disease (CD) is mucosal healing (MH). MH is predictive of reduced subsequent disease activity and clinical upset, and decreased need for further active treatment [1,2,3]. Several modalities are used in assessing overall disease activity and MH in CD. For instance, faecal calprotectin (FC) is a simple, non-invasive, and readily available tool; however, its accuracy in evaluating active small-bowel (SB) mucosal lesions in CD has often been debated [4]. Although cross-sectional imaging has been traditionally used in the evaluation of SB CD [5,6], endoscopy remains the ‘gold standard’ for assessing SB MH because it provides direct and clear observation of the SB mucosa.

Capsule endoscopy (CE) enables physicians to visualize the SB in a non-invasive manner. Hence, CE allows detection of SB mucosal lesions, as well as linear ulceration and luminal stenosis [7]. Recently, Esaki et al. [8] reported that CE enables the identification of SB damage in 88% of patients with established SB CD. Capsule retention is a potentially serious complication of CE; however, the rate of this complication decreases substantially if gastrointestinal (GI) patency is assessed prior to performing regular CE [9,10]. Consequently, CE is recommended as the initial diagnostic modality for SB assessment in patients with suspected CD and negative ileocolonoscopy [11].

To quantify/categorize SB inflammation, Gralnek et al. [12] developed a CE index, the Lewis score (LS), comprising 3 parameters: villous oedema, mucosal ulcer(s), and luminal stenosis. A LS < 135 indicates normal or insignificant mucosal inflammation, LS 135–790 indicates mild mucosal inflammation, and LS ≥ 790 indicates moderate-to-severe inflammation. LS has been validated in the diagnosis and follow-up of established CD [13]. Stratifying SB inflammatory activity at the time of diagnosis has relevant prognostic value in patients with isolated SB CD [14]. A CE-based assessment of SB MH is reported to be useful for predicting long-term clinical remission in CD [15]. Moreover, several retrospective studies have highlighted the potential effect of CE on the therapeutic management of patients with established CD [16,17,18].

However, a high percentage of patients in clinical remission have findings suggestive of on-going inflammatory activity, as evidenced by C-reactive protein (CRP) and FC levels, CE, and cross-sectional imaging [19]. Kopylov et al. [20] demonstrated that a positive CRP level was found in 30.8% of patients with CD in clinical remission, while a high LS on CE was found in 84.6%. Nevertheless, patients with CD who are clinically asymptomatic may not wish to receive additional treatment, and/or physicians may be reluctant to recommend additional treatment in such situations.

Therefore, the aim of this prospective study was to evaluate using CE additional treatment in patients with inflammatory activity, regardless of the presence of clinical symptoms.

## 2. Patients & Methods

### 2.1. Patients

This prospective, multicenter study was conducted in hospitals affiliated with the Department of Gastroenterology & Hepatology at Nagoya University Graduate School of Medicine. Key inclusion criteria were patients with established CD who were >10-years-old and scheduled to receive a PillCam patency capsule (PPC), and thereafter CE. For patients to be eligible for inclusion, the colon had to be clear of any inflammation, as confirmed using a conventional colonoscopy. Key exclusion criteria were the presence of any contraindications to anti-tumor necrosis factor (anti-TNF) agents and/or those who were considered non-appropriate candidates by their physician.

### 2.2. Study Protocol

**Figure 1:**
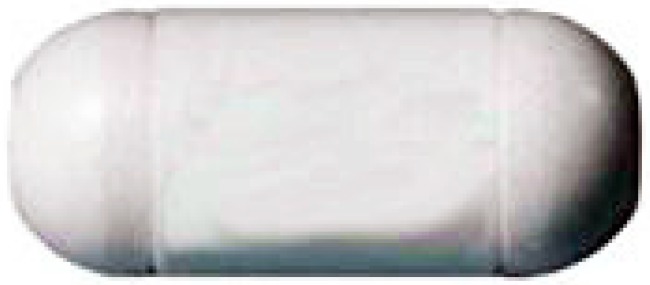
PillCam patency capsule.

The study protocol involved two rounds of CE. Patients with CD referred for CE evaluation were informed of the study, aims, and its protocol. Once consent was obtained, patients who accepted to participate underwent the baseline CE at their local hospital. For every patient who was considered for study inclusion, the absence of colonic inflammation, confirmed by a conventional colonoscopy, was necessary. PPC was used before CE, including follow-ups, in all patients who agreed to participate. The PPC consists of lactose and 10% barium, which dissolves when intestinal fluids come into contact with them through a window at the edges of the PPC. PPC is similar to the second-generation Agile patency capsule, with the only difference being that the radiofrequency identification tag has been removed [10] (Figure 1). GI patency was evaluated either by confirming excretion of an intact PPC or by obtaining a plain abdominal X-ray and/or computed tomography (CT), generally between 30 and 33 h after PPC ingestion. The capsules used for CE were PillCam^®^SB2 or SB3 (manufactured by Medtronic, Minneapolis, MN, USA), which measure 26 × 11 mm and are propelled by peristalsis. All subjects whose GI patency was confirmed underwent CE, at their earliest convenience, following an overnight fast without prokinetics or prior laxative bowel preparation. Patients whose GI patency was not confirmed were excluded from further participation in this study. Following anonymization, the CE videos were sent to Nagoya University Hospital via the Nagoya network system, as described in References [21,22]. Two expert CE readers (MN, TY) independently reviewed each CE and calculated the LS. They also read the second CE videos and remained blinded to the treatment provided following the baseline CE reports. SB cleansing was evaluated in four grades according to previous literature [23,24]. In cases of any discordance in LS results, the experts discussed all relevant CE images and provided final LS by consensus agreement. The report of each CE was then sent back to the local hospital and further clinical management was left with the treating physician/team. Clinical data related to PPC and CE, including adverse events, were collected.

Clinical remission was defined as a CD activity index (CDAI) score < 150. Since a LS < 135 can occasionally be associated with the presence of aphtha(e) or villous/fold oedema on CE, only a LS =0 was defined as complete MH. If LS ≤ 135 on baseline CE, suggestive of normal or clinically insignificant mucosal inflammatory change [25], the patient finished the study (non-active CD group). In the case of LS > 135, the treating teams could proceed with additional treatment, regardless of the presence of any clinical symptoms (active CD group). For patients of the active group, the follow-up CE was scheduled 6 months later, and it was performed with the same protocol as the baseline one.

### 2.3. Evaluations

Comparison was made between the clinical background and the baseline CE findings between the active and non-active groups. Additional therapeutic effects were evaluated in the active group, especially in asymptomatic patients, who underwent follow-up CEs. Primary outcome was any reported change in CE findings between the two CE procedures. Key secondary outcomes were changes in the CDAI and CRP level at the time of follow-up CE (for the active group), as well as any adverse events of PPC and CE procedures.

### 2.4. Statistical Analysis

The statistical software package SPSS for Windows (SPSS Inc., Chicago, IL, USA) was used for data analysis. The Wilcoxon signed-rank test was used to compare the LS based on CE and changes in each marker before and after any additional treatment. Patients’ demographic data at the baseline examination was compared between the active and non-active groups using the Mann-Whitney U test or *χ*^2^ test. In all analyses, a *p* value < 0.05 was considered statistically significant.

### 2.5. Ethical Considerations

This study was approved by the ethics Committee of Nagoya University Hospital. This study was registered in the University Hospital Medical Information Network, a clinical trials registry (UMIN000008486). Written informed consent was obtained from all participants.

## 3. Results

**Figure 2:**
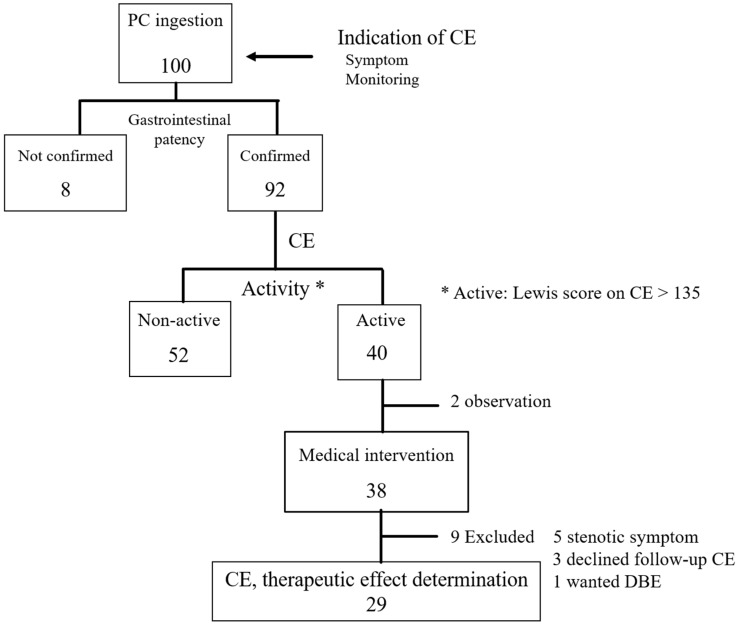
Flow chart of present study. Abbreviations PC, patency capsule; CE, capsule endoscopy; DBE, double balloon enteroscopy.

Between September 2012 and April 2016, 100 patients were referred for CE and approached for inclusion in this study at the Nagoya University Hospital and its 5 affiliated hospitals, as shown in Figure 2. Of these patients, 92 had confirmed GI patency and were eventually included in this study. Absence of colonic inflammation was confirmed, prior to study entry, with a conventional colonoscopy which was performed at a median of 14 (range 1–29) months prior to inclusion in the study.

In the 92 baseline CEs, the grades of SB cleansing (for the whole SB) were excellent, good, fair, and poor in 19, 59, 12, and 2 patients, respectively. Of 92 patients, 40 (43.4%) had findings of active CD (*active CD group*); clinically, 28/40 patients were symptomatic. Their symptoms were diarrhea (*n* = 20), abdominal pain (*n* = 4), bloody stool (n = 3), and abdominal fullness (*n* = 1). The remainder (*n* = 52), with LS < 135 on baseline CE, comprised the *non-active CD group*. CDAI scores were not significantly different between the two groups; however, LS was significantly higher in the active group than in the non-active CD group. Hemoglobin and serum albumin levels were significantly lower in the active group than in the non-active CD group (Table 1).

We defined as regular use of non-steroidal anti-inflammatory drugs (NSAIDs), the use of this class of medications for more than 6 months irrespective of type and dose. This was clarified by review of the medical charts and patient interview. None of the patients regularly had used NSAIDs before and/or after CEs. Of 38/40 patients of the active CD group who received additional anti-inflammatory treatment(s) (infliximab, *n* = 8; 5-aminosalicylic acid, *n* = 7; azathioprine, *n* = 6; adalimumab, *n* = 4; elemental diet, *n* = 2; prednisolone, *n* = 1; and mercaptopurine, *n* = 1), 29/40 (72%) underwent follow-up CEs to assess the therapeutic effect on MH.

Following additional treatment for 6 months, the mean LS improved from 691 ± 126 to 394 ± 99. Villous oedema before and after treatment was detected in 20 and 11 patients, respectively. Of all the 29 patients who had ulcers at baseline CE, 19 improved and 4 no longer had an ulcer. The LS of 4 patients who had a baseline LS > 1000 decreased; however, only 2/29 patients achieved MH (Table 2). The mean CDAI score and LS significantly improved 6 months after the start of additional treatment (Table 3), although the mean CRP level did not improve dramatically.

**Figure 3:**
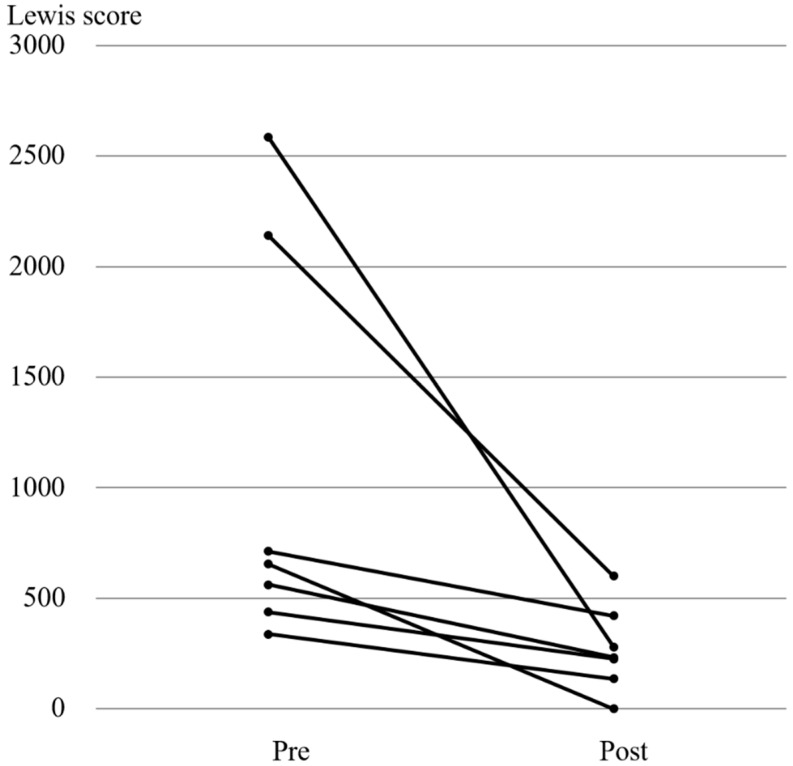
Changes in the Lewis scores in patients who received biologics as additional treatment. Abbreviations Pre, pre-treatment; post, post-treatment.

**Figure 4:**
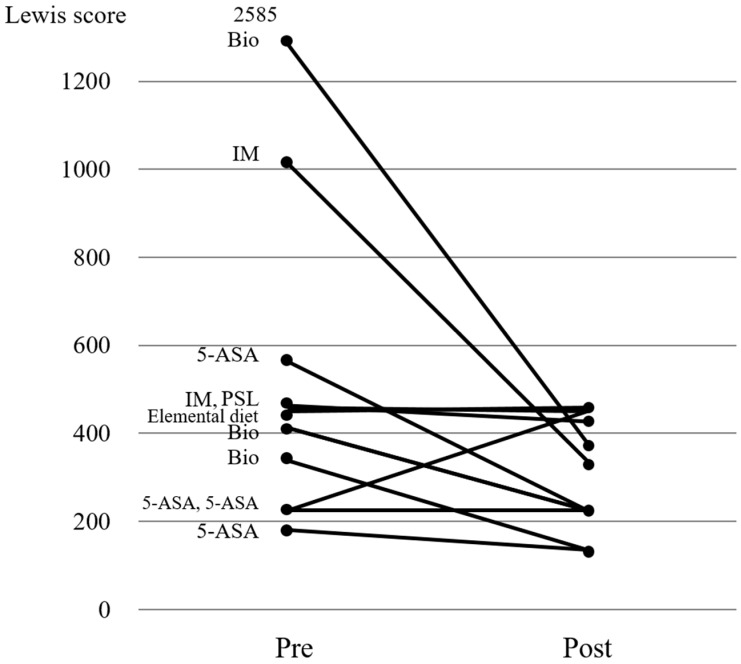
Changes in the Lewis scores in asymptomatic patients before and after treatment. Bio, biologic; IM, infliximab; 5-ASA, 5-aminosalicylic acid; PSL, prednisolone; pre, pre-treatment; post, post-treatment.

All 7 patients who received additional treatment with biologics had improvement of LS on repeat CE (Figure 3). Of the 11 patients who were asymptomatic, 4 received 5-ASA, 3 received biologics, 2 received immunomodulators, 1 received prednisolone, and 1 received elemental diet as additional treatment (Figure 4). LS improvement was noted in 8/11 (72.7%) patients; however, none of them achieved MH. No adverse events occurred throughout the study, as no retention of PPC or capsule was noted. Of 29 patients who received additional treatment and underwent follow-up CE, 23 patients’ clinical course could be confirmed post-study in November 2017. During the median follow-up 36 months, 12 patients did not undergo change in treatment, although 10 patients were given additional treatment and 1 underwent surgery.

## 4. Discussion

Our study confirms LS improvement in 23/29 patients (79.3%), irrespective of symptoms status, who received additional treatment for active CD based on the findings of baseline CE. Interestingly, 8/11 asymptomatic CD patients (72.7%) had improvement in their LS, regardless of the type of additional treatment provided. These results underline a couple of key points. First, asymptomatic CD patients may require additional treatment or simply increasing the dose of their existing therapeutic regimen to achieve MH. Additional medication had a positive effect in both asymptomatic and symptomatic CD patients, as shown in Figure 2, Figure 3 and Figure 4. Second, CE is a valid tool in evaluating both therapeutic effect, as well as confirming MH in patients with asymptomatic (active) CD.

In CD, it remains controversial whether the physician should set the therapeutic target at MH or clinical remission. If the therapeutic goal is MH rather than clinical (symptoms) remission, this can only be evaluated by endoscopy. The POCER study suggested that monitoring using early colonoscopy and treatment step-up was better in preventing postoperative CD recurrence, than conventional drug therapy alone [26]. On the other hand, Kim et al. reported that endoscopic monitoring did not significantly contribute to the non-hospitalization rate associated with CD, compared with ulcerative colitis [27]. However, the definition of MH remains unclear. Total disappearance of mucosal ulcerations has the advantage of providing irrefutable evidence of MH, but this strict ‘black-and-white’ goal may be difficult to achieve [28]. For instance, a patient with numerous deep mucosal ulcerations in whom treatment leads to healing of all but a superficial mucosal ulceration will be classified as a ‘non-responder’.

To date, only few studies have shown the necessity and effectiveness of additional treatment for CD patients in clinical remission and endoscopically-confirmed active lesions, although the significance of monitoring has been widely recognized. Once the therapeutic effects for such patients have been clarified, physicians may recommend a change of the medication regimen. Under the notion of treat-to-target, Ungar et al. suggested a significant association between serum levels of anti-TNF agents and the level of mucosal healing as the therapeutic goal [29]. Physicians may modify the dose of biologic treatment with reference to the serum levels even if patients have no symptoms. The CALM study demonstrated the significance of tight control, including timely escalation with anti-TNF therapy. Combining clinical symptoms with biomarkers in patients with early CD results in better clinical and endoscopic outcomes than symptom-driven decisions alone [30]. This study also supported the importance of tight control in CD by evaluating treatment outcomes in asymptomatic patients.

The development and introduction of a new, more effective drug will provide better CD monitoring. Endoscopic findings may become the best way to evaluate MH in CD because endoscopy provides direct visualization of the intestinal mucosa and enables physicians to detect even subtle mucosal lesions. However, it is unclear whether small lesions affect the long-term clinical outcome in CD. Moreover, it is questionable whether physicians can determine MH in patients with small lesions. LS calculation does not include the evaluation of such lesions. Therefore, MH according to LS means that there is no mucosal ulcer, but this does not preclude the presence of erosions or mucosal aphtha(e). Niv score or CECDAI, on the other hand, classifies erosion and small ulcers <5 mm under the same category [31,32]. It may be necessary to evaluate the outcome of patients with and without erosions.

If physicians can ignore small lesions to consider the long-term outcome of CD, abdominal ultrasonography and magnetic resonance enterography (MRE) will also be excellent modalities to evaluate MH. Koulaouzidis et al. reported that the LS appears to have only a fair correlation with the FC level, as well as other serological markers of inflammation [33]. FC level does not seem to be a reliable biomarker for significant SB inflammation. Nevertheless, an FC level ≥ 76 µg/g may be associated with appreciable visual inflammation on SB CE in patients with a prior negative diagnostic workup, and it may become the surrogate marker of CD.

This study has few limitations. The overall sample size of this study was small. This study focused on the Lewis score for next treatment, and laboratory data or calprotectin was not collected on follow-up. Of the patients with active CD based on CE, the percentage of patients who received an intervention was low because five patients had developed small-bowel stenosis, as demonstrated on a CT scan. The others did not wish to receive additional treatment. The kind of intervention depended on the physician and patients according to the protocol. Therefore, the balance between the degree of CD activity and kind of medication may not always be suitable. In conclusion, this prospective, multi-center study demonstrated that additional treatment regardless of clinical remission reduces mucosal inflammation in CD.

## Figures and Tables

**Table 1:** Demographic data of the patients at the point of study inclusion.

	Total	Active Group	Non Active Group	*p* Value
*N*	92	40	52	
Age, mean ± SD, years old	37.2 ± 12.3	37.5 ± 12.3	37.1 ± 12.9	0.8624
Gender, M/F	68/24	29/11	39/13	0.9751
BMI	21.3 ± 3.2	21.5 ± 3.6	21.1 ± 2.9	0.603
Duration of disease, mean ± SD, months	117.1 ± 96.7	93.9 ± 73.3	135 ± 108.8	0.093
Montreal classification				
Age at diagnosis				
<17 years	7	3	4	
17–40 years	70	30	40	
>40 years	15	7	8	
Location				
L1—ileal	38	17	21	
L2—colonic	0			
L3—ileocolonic	54	23	31	
Behavior				
B1—Non-stricturing, non-penetrating	72	28	44	
B2—Stricturing	15	9	6	
B3—Penetrating	5	3	2	
p-perianal disease	9	6	3	
History of GI surgery	53/92	25/40	28/52	0.5353
Ileo-colonic resection	26	12	14	
Ileal resection	21	9	12	
Ileo-colonic resection plus Ileal resection	4	3	1	
Colonic resection	2	1	1	
Any symptom	28/92	19/40	9/52	0.0038
CDAI	104 ± 56	116 ± 72	95 ± 39	0.277
Laboratory data				
CRP (mg/dL), mean ± SD	0.36 ± 0.62	0.53 ± 0.75	0.24 ± 0.47	0.0761
Hb (g/dL), mean ± SD	13.3 ± 2.1	12.7 ± 2.5	13.8 ± 1.6	0.0201
Albumin (g/dL), mean ± SD	4.0 ± 0.5	3.9 ± 0.5	4.2 ± 0.4	0.0014
Indication of CE				
Symptom(s)	28	19	9	
diarrhea	20	15	5	
abdominal pain	4	2	2	
bloody stools	3	2	1	
abdominal fullness	1	0	1	
Monitoring	64	21	43	
PPC and CE				
PPC intact body excretion	62/92	26/40	36/52	0.8377
Gastric transit time (min.)	45.5 ± 42.4	45.6 ± 40.3	47.2 ± 44.3	0.9904
SBTT (min.)	248.8 ± 128.8	270.7 ± 149.5	231.7 ± 108.5	0.3042
Lewis score, mean ± SD	396 ± 706	844 ± 892	52.1 ± 66.1	<0.0001
Treatment				
Anti TNF-α agent	54/92	23/40	31/52	
5-ASA	78/92	35/40	43/52	
Immunomodulator	14/92	5/40	9/52	
Elemental diet	57/92	23/40	34/52	

**Table 2:** 29 cases where intervention.

Case	Medicine	Intervention	Lewis Score							
			Total Score		Villous Edema		Ulcer		Stenosis	
			Pre	Post	Pre	Post	Pre	Post	Pre	Post
1	Infliximab	dose up 5 ⇒ 10 mg/kg	2914	2824	112	112	450	360	2352	2352
2	Infliximab	introduction 5 mg/kg	2585	280	8	0	225	0	2352	280
3	Adalimumab	introduction 160 mg	2140	600	340	0	1800	600	0	0
4	Azathiopurin	introduction 50 mg	1012	337	112	112	900	225	0	0
5	mercaptopurine	introduction 20 mg	900	1368	0	168	900	1200	0	0
6	5-ASA	dose up 3000 ⇒ 4000 mg	712	712	112	112	600	600	0	0
7	Infliximab	introduction 5 mg/kg	712	421	112	0	600	225	0	196
8	Adalimumab	introduction 160 mg	654	0	204	0	450	0	0	0
9	Adalimumab	introduction 160 mg	600	0	0	0	600	0	0	0
10	Infliximab	dose up 5 ⇒ 10 mg/kg	562	196	112	0	450	0	0	196
11	5-ASA	dose up 1500 ⇒ 2000 mg	562	225	112	0	450	225	0	0
12	5-ASA	introduction 3000 mg	562	337	112	112	450	225	0	0
13	Infliximab	introduction 5 mg/kg	562	233	112	8	450	225	0	0
14	Azathiopurin	introduction 50 mg	504	180	204	0	300	180	0	0
15	Prednisolone	introduction 20 mg	458	450	8	0	450	450	0	0
16	5-ASA	dose up 2000 ⇒ 3000 mg	458	147	8	12	450	135	0	0
17	Azathiopurin	introduction 50 mg	450	458	0	8	450	450	0	0
18	Adalimumab	introduction 160 mg	436	225	136	0	300	225	0	0
19	Elemental diet	dose up	429	225	204	0	225	225	0	0
20	Elemental diet	dose up	412	225	112	0	300	225	0	0
21	Infliximab	dose up 5 ⇒ 10 mg/kg	412	225	112	0	300	225	0	0
22	Infliximab	introduction 5 mg/kg	337	135	112	0	225	135	0	0
23	Azathiopurin	introduction 50 mg	300	278	0	8	300	270	0	0
24	Azathiopurin	dose up 50 ⇒ 75 mg	300	180	0	0	300	180	0	0
25	Azathiopurin	introduction 50 mg	233	143	8	8	225	135	0	0
26	Infliximab	dose up 5 ⇒ 10 mg/kg	225	233	0	8	225	225	0	0
27	5-ASA	dose up 2000 ⇒ 3000 mg	225	225	0	0	225	225	0	0
28	5-ASA	dose up 1500 ⇒ 3000 mg	225	450	0	0	225	450	0	0
29	5-ASA	dose up 2000 ⇒ 3000 mg	180	135	0	0	180	135	0	0

**Table 3:** Biomarker levels at baselines and 6 months later.

	Pre-Treatment	Post-Treatment	*p* Value
CDAI	102 (5–253)	68 (0–231)	0.0057
Lewis score	458 (180–2914)	233 (0–2824)	0.0004
CRP level (mg/dL)	0.55 ± 0.80	0.30 ± 0.51	0.0652
WBC count (/µL)	6822 ± 2602	5920 ± 1807	0.1663
Hb level (g/dL)	12.7 ± 2.5	13.2 ± 1.9	0.7843
Plt count(×1000/mm^3^)	26.6 ± 6.1	26.7 ± 7.0	0.5014
Albumin level (g/dL)	3.9 ± 0.6	4.1 ± 0.5	0.1297

Data are presented as a mean ± standard deviation. CDAI, Crohn’s disease activity index; CRP, C-reactive protein; WBC, white blood cell; Hb, hemoglobin; Plt, platelet.

## Abbreviations

5-aminosalicylic acid (5-ASA), C-reactive protein (CRP), Crohn disease (CD), Crohn’s disease activity index (CDAI), capsule endoscopy (CE), double-balloon endoscopy (DBE), faecal calprotectin (FC), gastrointestinal (GI), Lewis score (LS), mucosal healing (MH), non-steroidal anti-inflammatory drugs (NSAIDs), PillCam patency capsule (PPC).
